# Wheat Type One Protein Phosphatase Participates in the Brassinosteroid Control of Root Growth via Activation of BES1

**DOI:** 10.3390/ijms221910424

**Published:** 2021-09-27

**Authors:** Mariem Bradai, Vitor Amorim-Silva, Nibras Belgaroui, Alicia Esteban del Valle, Marie-Edith Chabouté, Anne-Catherine Schmit, Rosa Lozano-Duran, Miguel Angel Botella, Moez Hanin, Chantal Ebel

**Affiliations:** 1 Laboratory of Biotechnology and Plant Improvement, Center of Biotechnology of Sfax, BP “1177”, University of Sfax, Sfax 3018, Tunisia; mariem@psc.ac.cn; 2 Plant Physiology and Functional Genomics Research Unit, Institute of Biotechnology of Sfax, BP “1175”, University of Sfax, Sfax 3038, Tunisia; nebrasbelgaroui@yahoo.fr (N.B.); moez.hanin@isbs.usf.tn (M.H.); 3 Shanghai Center for Plant Stress Biology, CAS Center for Excellence in Molecular Plant Science, Chinese Academy of Sciences, Shanghai 201602, China; lozano-duran@sibs.ac.cn; 4 Departamento de Biología Molecular y Bioquímica, Instituto de Hortofruticultura Subtropical y Mediterranea “La Mayora”, Universidad de Malaga-Consejo Superior de Investigaciones Científicas (IHSM-UMA-CSIC), Universidad de Málaga, Campus Teatinos, 29071 Málaga, Spain; vitorsergiosilva@gmail.com (V.A.-S.); aesteban@uma.es (A.E.d.V.); mabotella@uma.es (M.A.B.); 5 Institut de Biologie Moléculaire des Plantes, CNRS, Université de Strasbourg 12, rue du Général Zimmer, 67084 Strasbourg, France; marie-edith.chaboute@ibmp-cnrs.unistra.fr (M.-E.C.); anne-catherine.schmit@ibmp-cnrs.unistra.fr (A.-C.S.)

**Keywords:** type one protein phosphatase, PP1, Brassinosteroids, signaling, root, meristem, BES1

## Abstract

Brassinosteroids (BRs) play key roles in diverse plant growth processes through a complex signaling pathway. Components orchestrating the BR signaling pathway include receptors such as kinases, transcription factors, protein kinases and phosphatases. The proper functioning of the receptor kinase BRI1 and the transcription factors BES1/BZR1 depends on their dephosphorylation by type 2A protein phosphatases (PP2A). In this work, we report that an additional phosphatase family, type one protein phosphatases (PP1), contributes to the regulation of the BR signaling pathway. Co-immunoprecipitation and BiFC experiments performed in Arabidopsis plants overexpressing durum wheat *TdPP1* showed that *TdPP1* interacts with dephosphorylated BES1, but not with the BRI1 receptor. Higher levels of dephosphorylated, active BES1 were observed in these transgenic lines upon BR treatment, indicating that *TdPP1* modifies the BR signaling pathway by activating BES1. Moreover, ectopic expression of durum wheat *TdPP1* lead to an enhanced growth of primary roots in comparison to wild-type plants in presence of BR. This phenotype corroborates with a down-regulation of the BR-regulated genes CPD and DWF4. These data suggest a role of PP1 in fine-tuning BR-driven responses, most likely via the control of the phosphorylation status of BES1.

## 1. Introduction

Brassinosteroids (BRs) are phytohormones involved in diverse developmental and physiological responses going from root development, cell division and cell elongation, to photomorphogenesis and seed germination (for review see [1,2]). These BR-triggered responses occur through a well-defined signaling pathway starting with BR perception by the extracellular LRR domain of the BRI1 (BRASSINOSTEROID-INSENSITIVE 1) receptor [3,4] at the cell surface together with its co-receptor BAK1 (BRI1-ASSOCIATED RECEPTOR KINASE 1/SERK3 (SOMATIC EMBRYOGENESIS RECEPTOR KINASE [1,5,6]. In BR signaling, several transphosphorylation events mediated through BSK1 (BRASSINOSTEROID-SIGNALLING KINASE1), CDG1 (CONSTITUTIVE DIFFERENTIAL GROWTH1) and BIN2 (BRASSINOSTEROID INSENSITIVE 2) kinases will lead to the activation of BSU1 (BRI1-SUPPRESSOR1) phosphatase, which acts to dephosphorylate and inactivate BIN2, the negative regulator of BR signaling. At low BR concentrations, BIN2 phosphorylates two homologous transcription factors, BZR1 (BRASSINAZOLE-RESISTANT1) and BR-INSENSITIVE-EMS-SUPPRESSOR1 (BES1/BZR2) (hereafter referred to as BES1), and inhibits their nuclear localization and DNA-binding activity [7]. The cytoplasmic retention of the inactivated BES1/BZR1 is promoted by 14-3-3 protein binding (for review see [8]). At higher BR levels, along with the BIN2 inactivation, type 2A protein phosphatase PP2A activates BZR1/BES1 via their dephosphorylation to develop appropriate cell responses and attenuates cellular BR biosynthesis via a negative feedback mechanism down-regulating BR biosynthetic genes such as DWF4 and CPD [9,10]. In addition, PP2A can also reversibly inactivate BRI1 through dephosphorylation [11].

The *Arabidopsis thaliana* (hereafter referred to as Arabidopsis) genome encodes around 150 different protein phosphatases grouped in three major classes depending on their phospho-substrates (phospho-serine, phospho-tyrosine and double-specificity S/T phosphatases; for review see [12,13]). Type one protein phosphatases (TOPP/PP1) are particular serine/threonine protein phosphatases, since their phosphatase activity is driven by interactors that control their activity, substrate specificity or subcellular localization [13,14]. Arabidopsis contains nine TOPP isoforms (called TOPP1-9), while rice, *Vicia faba* and bread wheat have 5, 4 and 18 PP1s, respectively [13,15,16,17]. Beyond an extremely strong primary structure conservation, PP1s are characterized by their functional diversity as they participate in multiple biological processes such as cell cycle regulation, glucose metabolism, transcription, protein synthesis, plant development, light signaling and stress responses [12,13]. The analysis of the Arabidopsis dominant-negative mutant *topp4-1* revealed a role of TOPP4 in the gibberellic acid (GA) pathway by DELLAs dephosphorylation, and its subsequent degradation by the 26S proteasome [18] in stopping PIF5 (PHYTOCHROME-INTERACTING FACTOR5) degradation during light signaling [19], and in dephosphorylating PIN1 to regulate auxin gradient at the epidermis [20]. Lately, TOPP4 has also been shown to be involved in plant autoimmunity, probably by its binding to the NB-LRR receptor SUT1 [21,22]. TOPP1 reinforced by its interaction with Arabidopsis Inhibitor 2 (AtI2), considered as a negative regulator of TOPP activities [23], is involved in ABA signaling by inactivating SnRK2 kinase [24]. TOPP9 and TOPP8, also named, respectively, AUN1 and AUN2, have been shown to regulate cell wall integrity during pollen tip growth and root hair elongation [25]. In rice, the RSS1 (RICE SALT SENSITIVE 1) protein interacts with OsPP1a to control meristem maintenance upon salt stress [17,26], and OsPP1a overexpression confers salinity tolerance to rice plants [27].

Functional characterization of PP1 in plants is hampered by high redundancy and versatility, and furthermore by the large diversity of PP1-interacting proteins (PIP) that are essential to drive substrate specificity, activity and/or subcellular localization [14,28]. The durum wheat *TdPP1a* (hereafter referred to as *TdPP1*) is part of a large multigenic family in wheat composed of 18 different genes located on the different subgenomes and is phylogenetically closely related to *Arabidopsis* TOPP4, with 88% identity at the protein level [16]. Being the only wheat PP1 characterized so far, *TdPP1* was shown to be strongly expressed in durum wheat roots, an active phosphatase in vitro and to be able to interact with AtI2 [16], but still, its biological function in planta remains unknown.

Therefore, the current research aims to characterize the function of the durum wheat PP1, through its ectopic expression in Arabidopsis. Our results provide evidence for an original contribution of *TdPP1* in root growth and BRs signaling via activation of the BES1 transcription factor.

## 2. Results

**Figure 1:**
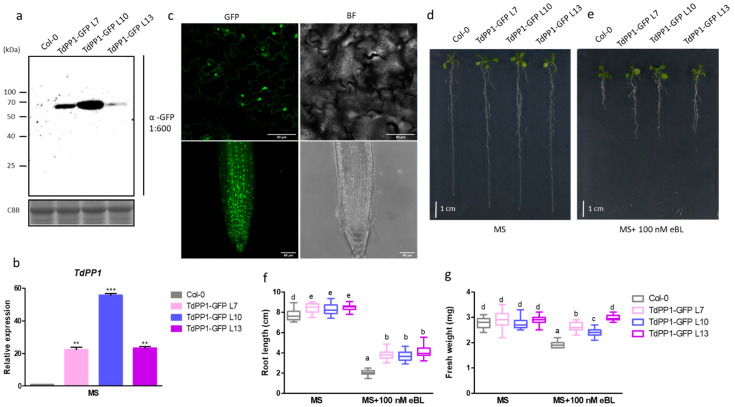
Arabidopsis *TdPP1* overexpression promotes root growth in the presence of eBL. (**a**) TdPP1-GFP is expressed at different levels in transgenic Arabidopsis lines. Western blot using anti-GFP antibody (α-GFP) on total proteins extracted from WT and three different transgenic TdPP1-GFP (L7, L10 and L13) lines grown for 7 days in half-strength MS medium. CBB: Coomassie Brilliant Blue. (**b**) Analysis of *TdPP1* expression in Arabidopsis WT plants, and *TdPP1* OE (L7, L10 and L13). These plants were grown on half MS medium for 7 days, seedlings were collected and the expression level of the *TdPP1* was quantified by qRT-PCR using *ACT2* and *GAPC2* gene as a reference gene and normalized to the expression of *ACT2.* (**c**) TdPP1-GFP is ubiquitously localized in Arabidopsis epidermal cells and roots of TdPP1-L10. Representative confocal images of 5-day-old TdPP1-GFP L10. Images show GFP fluorescence in green and cells/roots in bright field (BF). (**d**,**e**) TdPP1-GFP transgenic (L7, L10 and L13) lines have longer roots in the presence of exogenous eBL. Representative pictures of seedlings grown for 4 days in half-strength MS and transferred to half-strength MS without (**d**) or with 100 nM eBL (**e**) for 8 days. Scale bar: 1 cm. (**f**) Root length and (**g**) fresh weight of WT Col-0 and transgenicTdPP1-GFP OE lines. Different letters indicate statistically significant differences between transgenic lines and WT determined by one-way ANOVA followed by Tukey’s multiple comparison test (*p*-value < 0.05). Data represent mean values, error bars are SD and *n* = 30 seedlings per experiment. The experiment was repeated at least three times with similar results. **：*p* < 0.01, ***: *p* < 0.001.

The durum wheat *TdPP1* [16] was ectopically expressed in the Arabidopsis Col-0 ecotype under the control of the CaMV 35S promoter with a GFP C-terminal fusion. Three homozygous lines (TdPP1-GFP L7, TdPP1-GFP L10, TdPP1-GFP L13) with a single insertion based on the segregation of the selectable marker, were selected for assessing transgene expression by RT-qPCR and immunoblot analyses. *TdPP1* is expressed in these three transgenic lines at various levels, with TdPP1-GFP L10 and TdPP1-GFP L13 being the highest and the lowest expressing lines, respectively (Figure 1a,b). Observations by confocal microscopy of TdPP1-GFP L10 roots showed that *TdPP1* is localized ubiquitously within the cell (Figure 1c), as it was previously reported in *Nicotiana bentamiana* [16].

Phenotypical analyses showed that the three lines have significantly longer primary roots compared to wild-type (WT) seedlings (Figure 1d). Previous observations of Arabidopsis plants overexpressing TOPP4 reported greater aerial tissue size but no data on roots were described [18]. Optimal primary root growth and development is orchestrated by a complex, fine-tuned interplay of different phytohormones [29]. Among these, brassinosteroids (BR) promote root growth at different levels along the root [30]. In our case, when exposed to 100 nM of 24-epibrassinolide (eBL), the three transgenic lines exhibited, indeed, 40% higher primary root length compared to WT (Figure 1d–f), concomitant to a 30% increase in fresh weight (Figure 1g).

**Figure 2:**
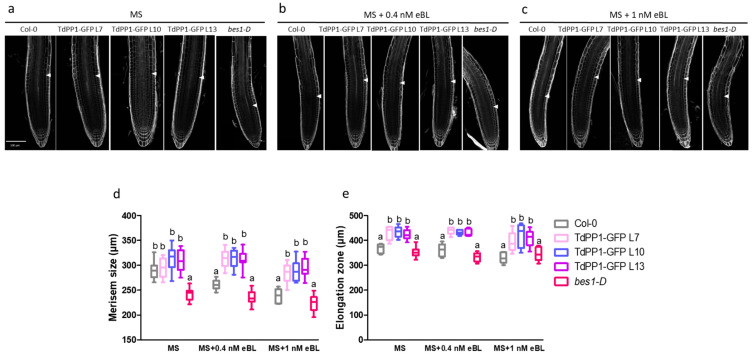
Roots of *TdPP1* expressing lines are hyposensitive to exogenous eBL. (**a**–**c**) Effect of exogenously applied eBL on meristem size. Confocal images of 5-day-old WT, TdPP1-GFP OE lines and *bes1-D* grown under control conditions (MS; **a**), or treated with 0.4 nM (**b**) or 1 nM eBL (**c**). Seedlings were treated with ClearSee, stained with calcofluor-white. Arrows indicate boundary between the proximal meristem and the elongation zone of the root. Scale bars: 100 µm. (**d**) Meristem size of WT, TdPP1-GFP transgenics and *bes1-D* exposed or not to eBL. Meristematic zone size was defined as the region of isodiametric cells from the QC up to the cell that was twice the length of the immediately preceding cell. Letters indicate statistically significant differences between WT vs TdPP1-GFP OE (L7, L10 and L13) and *bes1-D* as determined by one-way ANOVA followed by Tukey’s multiple comparison test (*p*-value < 0.05). Data represent mean values of 15 measurements and error bars are SD. (**e**) Size of elongation zones of WT, TdPP1-GFP transgenics and *bes1-D* exposed or not to eBL. Elongation zone was considered as the region going from the end of the meristematic zone to the beginning of differentiation zone (emergence of first root hair). Letters indicate statistically significant differences between WT vs TdPP1-GFP OE (L7, L10 and L13) and *bes1-D* as determined by one-way ANOVA followed by Tukey’s multiple comparison test (*p*-value < 0.05). Data represent mean values of 15 measurements and error bars are SD.

It is well established that external application of BR can affect root growth in two contrasting ways, depending on the concentrations. While low BR concentrations (≤0.04 nM eBL) trigger root growth, concentrations higher than 0.04 nM inhibit root growth [31]. This root growth arrest can be explained by the fact that high eBL concentrations might inhibit cell division in meristematic regions while promoting cell elongation and differentiation [31,32,33]. Therefore, we monitored meristem size in WT and the *TdPP1* transgenic lines after an exposure to eBL (0.4 and 1 nM) because higher eBL concentrations (100 nM) used previously (Figure 1) avoid precise observations of meristematic tissues due to intense root curling. We used also as a control the BR-insensitive *bes1-D*, a gain-of-function mutant where BES1 protein stability is increased leading to a constitutive activation of BR signaling [9], hence negatively affecting meristem size due to a premature cell cycle exit [31]. When grown on a control ½ MS medium, *TdPP1* overexpressing lines (OE) had comparable meristem size to that of WT, while bes1-D had a shorter meristem size (Figure 2b,e). Treatment with 0.4 or 1 nM of eBL did not further change the meristem size of the *bes1-D* mutant. However, the meristem of WT seedlings was 20% shorter in the presence of 0.4 nM eBL. Interestingly, in *TdPP1* OE lines the meristem size was not affected by this eBL concentration (Figure 2c,e). Treatment with 1 nM eBL, reduced even more significantly the meristem size of WT (up to 40% decrease vs the MS grown WT seedlings) while this reduction was limited to 20% in the meristems of the *TdPP1* OE lines (Figure 2d,e). This result clearly indicates that the meristems of *TdPP1* OE lines show a decreased sensitivity to BR.

We next compared the size of the elongation zones in the three *TdPP1* OE lines with WT and *bes1-D* mutant (Supplementary Figure S1). Under control conditions, *TdPP1* OE lines had elongation zones about 20% longer than that of WT and *bes1-D*. In the presence of 0.4 nM and 1 nM eBL, the elongation zone did not expand significantly in the *TdPP1* overexpressors compared to WT (10% increase), indicating again that these lines are less sensitive to exogenous BR.

**Figure 3:**
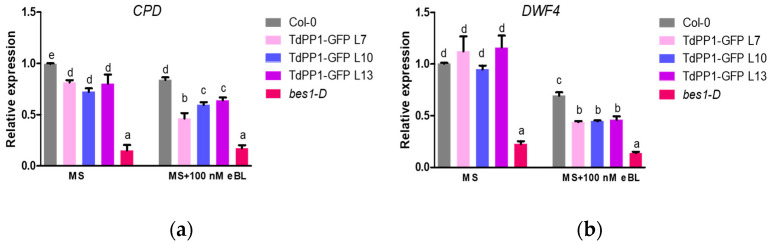
Relative expression of BR-biosynthesis genes *CPD* and *DWF4*. (**a**) Analysis of *CPD* and (**b**) *DWF4*, BR-responsive gene expression in Arabidopsis WT plants, TdPP1-GFP OE lines (L7, L10 and L13) and *bes1-D*. Plants were grown on half-strength MS medium for 7 days and transferred to half MS-strength liquid medium without or with 100 nM BL for 1 h. Seedlings were collected and the expression level of *CPD* and *DWF4* was quantified by qRT-PCR using the *ACT2* and *GAPC2* gene as reference genes. The expression of *CPD* and *DWF4* was normalized to the expression of *ACT2*. Data represent mean values and error bars are SD from the average of three biological replicates (10 seedlings per biological replicate). Letters indicate statistically significant differences between WT vs TdPP1-GFP OE (L7, L10 and L13) and *bes1-D* as determined by one-way ANOVA followed by Tukey’s multiple comparison test (*p*-value < 0.05).

To underpin the phenotype observed on the roots of the *TdPP1* overexpressors, we investigated the expression of the BR-biosynthesis CPD and DWF4 genes that are negatively regulated at high BR concentrations. For this purpose, CPD and DWF4 expression analyses were performed by qRT-PCR in the presence and absence of BR, on the TdPP1-transgenic lines along with WT, and *bes1-D* as controls. Our results show that under control conditions, *CPD* expression was slightly, but significantly, down-regulated in TdPP1-OE lines, while *DWF4* remained unchanged such as in WT (Figure 3). Treatment with eBL caused the down-regulation of both *CPD* and *DWF4* in WT seedlings (Figure 3), but this down-regulation was more pronounced in the *TdPP1* OE lines. It, therefore, indicates that *TdPP1* overexpression leads to an increased BR signaling.

**Figure 4:**
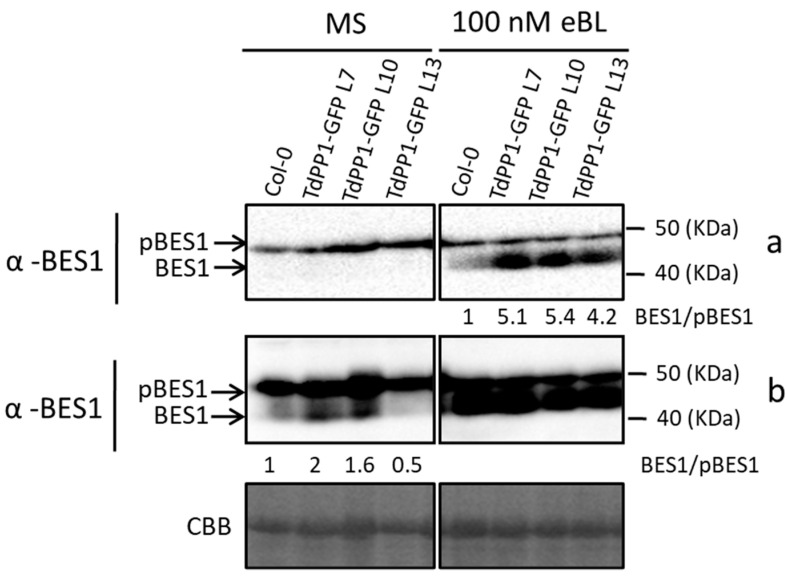
*TdPP1* overexpression interferes with the balance pBES1/BES1. Phosphorylation status of BES1 in TdPP1-GFP OE lines in response to exogenously applied eBL. Protein extracts of 7-day-old seedlings (WT, L7, L10 and L13) treated or not for 1 h with 100 nM eBL were analyzed by an immunoblot assay with a specific anti-BES1 antibody. Upper arrow indicates phosphorylated BES1 (pBES1) and lower arrow indicates dephosphorylated BES1 (BES1). (**a**) Standard exposure of the blot (**b**) exposure with increased sensitivity. CBB: Coomassie Brilliant Blue. Numbers below blots represent the ratio of unphosphorylated BES1 (BES1) to phosphorylated BES1 (pBES1).

To get insight into the molecular mechanism by which *TdPP1* contributes to BR signaling, we investigated whether *TdPP1* is able to interfere with the activity of the BES1 transcription factor. BES1 is a key protein in BR signaling that is regulated by PP2A phosphatases [1] and its dephosphorylation allows its nuclear translocation and activation [7]. To this end, we examined the levels and phosphorylation status of BES1 in *TdPP1* OE lines. Endogenous BES1 protein levels in WT and *TdPP1* transgenic lines under control conditions and after treatment with 100 nM eBL were analyzed by immunoblot using anti-BES1-specific antibody. As shown in Figure 4 (top panel), WT and *TdPP1* OE seedlings showed similar levels of phosphorylated BES1 in control conditions. Interestingly, treatment with 100 nM eBL increased the amount of dephosphorylated (active) BES1 in all three *TdPP1* lines compared with WT. When we used an ultra-sensitive detection system on the same blot (Figure 4, bottom panel b), we could observe an increased accumulation of dephosphorylated BES1 in *TdPP1* lines compared to WT seedlings already in control conditions.

**Figure 5:**
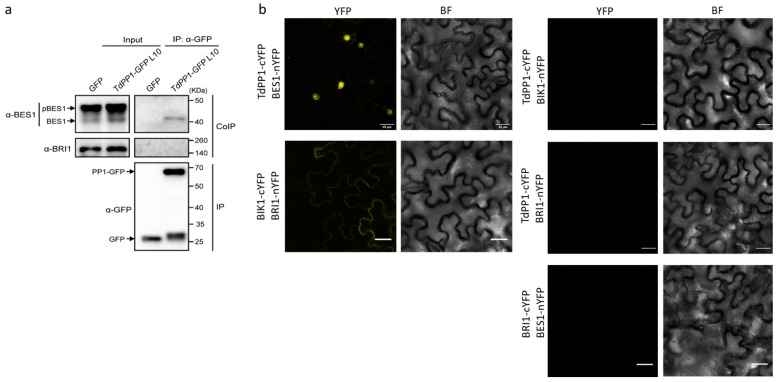
*TdPP1* binds to unphosphorylated BES1. (**a**) *TdPP1* co-immunoprecipitates with BES1 but not with BRI1. Total proteins were extracted from TdPP1-GFP L10 and 35S::GFP transgenic line as negative control. The protein extracts were immunoprecipitated using anti-GFP Trap beads. Total (input), immunoprecipitated (IP) and co-immunoprecipitated (CoIP) proteins were analyzed by immunoblotting. GFP was used as a negative control for Co-IP. TdPP1-GFP and GFP were detected with anti-GFP antibody (α-GFP), BES1 with anti-BES1 (α-BES1) antibody and BRI1 with anti-BRI1 (α-BRI1). The upper arrow indicates the band of the phosphorylated BES1 (pBES1), and the lower arrow indicates the band of dephosphorylated BES1 (BES1). (**b**) *TdPP1* interacts with BES1 and not with BRI1 in *Nicotiana benthamiana*. *N.benthamiana* leaves were co-agroinfiltrated with *Agrobacterium* strains expressing *TdPP1* protein fused to the C-terminus half of YFP and BES1 or BRI1 proteins fused to the N-terminus half of YFP and observed with confocal microscopy. Images show YFP fluorescence in yellow (YFP) and cells in bright field (BF). The proper expression of BRI1 was confirmed by positive interaction monitored as a fluorescence signal emitted by interaction between BRI1 and BIK1. Scale bars represent 25 μm.

Next, we investigated whether *TdPP1* interacts with BES1, which could account for its higher dephosphorylation in the transgenic lines. For this purpose, we performed co-immunoprecipitation (Co-IP) assays with the TdPP1-GFP L10 line using anti-GFP-Trap beads (Figure 5a). As a control we used BRI1 because, as with BES1, it is also dephosphorylated by PP2A [11]. Our result shows that TdPP1-GFP co-immunoprecipitated endogenous BES1, but not BRI1. Surprisingly, *TdPP1* only co-immunoprecipitated dephosphorylated BES1, despite its reduced proportion relative to the phosphorylated one in input (Figure 5a). These data strongly show that *TdPP1* is able to bind to and might dephosphorylate BES1.

We then decided to verify this TdPP1-BES1 interaction in planta by bimolecular fluorescence complementation (BiFC assays) in *N. benthamiana* leaves. Co-expression of the TdPP1-C-terminal half of YFP (cYFP) with the BES1-N-terminal half of YFP reconstituted a functional YFP protein in the nucleus with strong signals observed in the nucleolus (Figure 5b). This is in agreement with the Co-IP data, since most of the dephosphorylated BES1 is localized in the nucleus [9]. Consistent also with the Co-IP experiment, no interaction was found between *TdPP1* and BRI1 (Figure 5b) that interacts with BIK1 (BOTRYTIS-INDUCED KINASE 1), as previously shown [34]. Interestingly, in comparison to *TdPP1*’s ubiquitous localization throughout the transgenic cells (Figure 1), the BiFC data also show that *TdPP1* was able to relocate in the nucleus together with BES1. It is worth noting that *TdPP1* interaction is restricted to unphosphorylated BES1, as observed by co-IP, suggesting that PP1-pBES1 interaction is highly labile or that dephosphorylation activity of PP1 is highly performant, leading to a rapid BES1-PP1-complex nuclear translocation, likely observed by BiFC. Therefore, our data strongly suggest that, as with PP2As, *TdPP1* is able to bind BES1, thus causing its activation and nuclear translocation.

## 3. Discussion

Type one protein phosphatases are ubiquitous strongly conserved proteins with highly versatile activities still largely unknown in plants. Versatility is conveyed by high redundancy but also by their interaction with multiple regulators (PIPs) that modulate their activity or specify substrate binding or subcellular localization. The present study puts the light on a novel function for PP1s in promoting root growth through enhancing BR signaling. We showed here that wheat *TdPP1* contributes towards regulating BR signaling through activation of BES1.

Such a role in BR signaling pathway has never been reported so far for any plant PP1, in contrast to their known functions in other phytohormone signaling pathways. Indeed, Arabidopsis TOPP4 participates in GA signaling by dephosphorylation of DELLAs and in auxin transport by acting on PIN [18,20]. TOPP1 regulates ABA signaling by inhibiting SNF1-related protein kinases 2 (snRK2s; [24]), leading to partial release of the ABA-mediated root growth inhibition [24]. The latter example resembles to some extent our current observations, since besides PP2C, the main negative regulator of snRK2s [35,36], TOPP1 together with its regulatory partner AtI2, participates in snRK2s inhibition during ABA signaling [24]. *TdPP1* may act besides the trimeric PP2A in regulating BES1 activity, hence adding complexity but also flexibility in the BR signaling pathway. However, our data showed that in contrast to PP2A, *TdPP1* acts only on BES1 and has no visible effect on BRI1, suggesting that the wheat phosphatase may have a more subtle function in fine-tuning BR signaling. Alternatively, an association of *TdPP1* to the PP2A complex replacing its catalytic subunit (as both show significant similarity) is not excluded. Depending on their expression features, subcellular localization and interaction with their regulatory partners, PP1s may contribute at different levels to modulate BR signaling. Extensive redundancy of PP1s and high modularity of PIP counter, unfortunately, the deciphering of their multiple roles in biological processes.

Adequate root growth and meristem maintenance result from a complex intervention of different phytohormones with contrasting outcomes on particular cell types or root zones. Phytohormones such as auxins, cytokinins and BR regulate each other’s action, biosynthesis, transport and catabolism and have opposing effects on root growth [37,38]. The positive effect that *TdPP1* exerted on root growth upon BR exposure may be due to its capacity to induce subtle changes in BR signaling in a way that helps the plant to cope with the negative effects of high BR concentrations on root growth and development. Moreover, BR biosynthesis and active BES1 nuclear accumulation optima overlap in the elongation zone, and little or no nuclear active forms of BES1 are found in the root tip [39,40,41]. By binding to and activating BES1, under control conditions, *TdPP1* might slightly increase BR signaling through BES1 activation, all along the root, in a way that mimics optimal BR concentrations so that primary root growth is increased in these lines. Consistently also, the discrete down-regulation of CPD in the absence of BR may indicate that in untreated *TdPP1* OE plants, BR signaling might already be increased. By adding exogenous high concentrations of BR, *TdPP1* overexpressing lines are partially insensitive because with already longer roots they were better prepared to withstand high BR concentrations. Despite the increased activation of BES1 in transgenic lines, these lines do not accurately phenocopy the dominant negative effect of *bes1-D* on BR response, showing that in these lines BES1 can return to an inactive state.

Importantly, the overexpression of TOPP4 leads to Arabidopsis plants with greater plant heights, enlarged hypocotyl and leaves, probably because of an increase in GA signaling and responses [18]. TdPP1-overexpressing plants did not exhibit greater above-ground tissues but had slightly longer roots compared to WT, suggesting that *TdPP1* overexpression may not perturb GA signaling. It is possible, though, that *TdPP1* overexpression might affect the balance and signaling events of other hormonal pathways not yet observed, since BES1 is a hub for different hormonal signaling pathways, and that *TdPP1* may act on other phytohormone signaling components. Although BR signaling mechanisms in Arabidopsis and rice are well documented, involvement of PP1s in regulating the BR pathway was not reported before. In wheat, the major BR signaling components are conserved but highly redundant, with each component having one copy per subgenome [42]. Therefore, uncovering this novel role of PP1s on BES1 activation would have been difficult in polyploid wheat.

Altogether, the data presented herein highlight a new role for plant PP1s in BR signaling and open up new perspectives in manipulating BR pathway for crop improvement.

## 4. Materials and Methods

### 4.1. Plant Materials and Growth Conditions

Wild type Arabidopsis Col-0 (seeds from the NASC Stock Center) together with *bes1-D* mutant [9] and transgenic lines generated in the present study were grown as follows. After seed surface sterilization and stratification for 3 days at 4 °C, seedlings used for the brassinolide treatment, meristem size measurement, confocal imaging, quantitative RT-PCR (qRT-PCR) and phosphorylation status of BES1 were sowed on half strength Murashige et Skoog (½ MS) medium containing 1.5% sucrose and 0.8% agar. Plates were placed vertically or horizontally in a growth chamber with a photoperiod of 16 h light/8 h dark at 22 °C with 70% relative humidity. *Nicotiana benthamiana* seeds were sown directly on soil and cultured during 4 weeks at 22 °C with 70% relative humidity, with a photoperiod of 16 h light/8 h dark.

### 4.2. Transgenic Lines

The *TdPP1* ORF (KM203893) was PCR-amplified and cloned into the pENTR-D-TOPO vector using the pENTR Directional TOPO cloning kit (Invitrogen) as previously described by [16]. LR recombination was performed between the pENTR vector and the pB7FWG2 vector (Ref. [43], BASTA resistance) to create a C-terminal fusion of *TdPP1* with eGFP. This construct was mobilized into the Agrobacterium tumefaciens GV3101 strain for Arabidopsis Col-0 transformation using the floral dip method [44]. Selection rounds until T3 generation were performed on BASTA 10 µg/mL.

### 4.3. eBL Treatment and Meristem Size Measurement

For root length and fresh weight analyses, seedlings were grown vertically on ½ MS medium for 4 days prior to transfer to a fresh medium containing either mock (ethanol 80% as eBL solvent) or 100 nM eBL (PhytoTechnology Laboratories, Shawnee, KS, USA). After 8 days, seedlings were photographed and weighted. Root length was measured using Fiji [45] and graphs were performed using GraphPad Prism 6 (GraphPad Software, San Diego, CA, USA, www.graphpad.com). For meristem size, seedlings were grown for 5 days on ½ MS medium containing either mock (ethanol 80%), 0.4 nM or 1 nM eBL, then imaged and analyzed by confocal microscopy as described below.

### 4.4. Phosphorylation Status of BES1

Seedlings were grown vertically on ½ MS medium for 7 days prior to transfer to liquid ½ MS medium containing either ethanol 80% (eBL solvent) or 100 nM eBL for 60 min. Total proteins were extracted as described under “Extraction of Total Protein from Arabidopsis” and analyzed by immunoblotting using an anti-BES1 antibody (1:500; [46]) as described under “Western blotting”.

### 4.5. Western Blotting

One hundred mg of one-week-old Arabidopsis *TdPP1* OE seedlings were ground to powder in liquid nitrogen prior to the addition of 200 µL of 2× Laemmli Buffer and boiled at 95 °C for 10 min. After 5 min centrifugation at top speed in a regular bench centrifuge, the supernatant was loaded on 10% SDS-PAGE polyacrylamide gel and proteins were separated by electrophoresis. Transfer to PVDF membrane (Immobilon-P, Millipore, Burlington, MA, USA) was performed during one hour at 18 V using the Trans Blot SD semi-dry transfer cell (BioRad, Hercules, CA, USA). After blocking in 1× TTBS, 5% skimmed milk for 2 h and three washes in 1× TTBS, the primary anti-GFP antibody was added at a dilution of 1:600 (mouse anti-GFP clone B-2; Santa Cruz Biotechnology, Dallas, TX, USA) and incubated overnight at 4 °C. After three washes in 1× TTBS, the peroxidase-conjugated secondary antibody (anti-mouse IgG, 1/80,000; Sigma-Aldrich, St. Louis, MO, USA) was added during 1 h at room temperature. Detection was performed using the SuperSignal^®^ West Femto Maximum Sensitivity Substrate (Thermo SCIENTIFIC, Waltham, MA, USA) and images were acquired at different exposure times with Chemi-doc XRS+ System (BioRad).

### 4.6. Protein Extraction and Co-Immunoprecipitation (co-IP) Assays

Co-immunoprecipitation was performed as previously reported [47]. Briefly, total proteins were extracted from 100 mg of seedlings from Arabidopsis *TdPP1* OE line 10 (TdPP1-GFP L10) in an extraction buffer (2 mL/g of powder; 50 mM Tris-HCl, pH 7.5; 150 mM NaCl; 10% glycerol; 10 mM EDTA, pH 8; 1 mM NaF; 1 mM Na_2_MoO_4_·2H_2_O; 10 mM DTT; 0.5 mM PMSF; 1% (*v*/*v*) P9599 protease inhibitor cocktail (Sigma); Nonidet P-40, CAS: 9036-19-5 (USB Amersham life science) 0,5% (*v*/*v*)) at 4 °C for 30 min on constant end-over-end rocking. After a centrifugation (9000 rpm/min) for 20 min at 4 °C, supernatants were filtered by gravity through Poly-Prep Chromatography Columns (#731-1550 Bio-Rad), and 100 µL were saved as input for Western blot analysis. The remaining supernatants were incubated for 2 h at 4 °C with 15 µL GFP-Trap coupled with agarose beads (Chromotek). During incubation of protein samples with GFP-Trap beads, the final concentration of detergent (Nonidet P-40) was adjusted to 0.2% (*v*/*v*) in all cases to avoid unspecific binding to the matrix, as recommended by the manufacturer. Following incubation, the beads were collected and washed four times with a wash buffer (extraction buffer but without detergent). Finally, beads were resuspended in 75 µL of 2× Laemmli Sample Buffer and heated at 60 °C for 30 min to dissociate immunocomplexes from the beads. Total (input), immunoprecipitated (IP) and co-immunoprecipitated (CoIP) proteins were separated by electrophoresis in 10% SDS-PAGE, and analyzed as described by Western blot analysis using anti-BRI1 (1:2000; [48]), anti-BES1 (1:500; [46]) or anti-GFP (1:600; Santa Cruz Biotechnology) antibodies. Appropriate peroxidase-conjugated secondary antibodies were added as follows: anti-mouse IgG whole molecule Peroxidase (1:80,000; catalog No. A9044, Sigma-Aldrich) for anti-GFP, and anti-rabbit IgG whole molecule Peroxidase (1:80,000; catalog No. A0545, Sigma-Aldrich) for anti-BRI1 and anti-BES1.

### 4.7. Bimolecular Fluorescence Complementation (BiFC) Assays

The DNA constructs used for agroinfiltration were in pDEST-GW-VYNE for a N-terminal fusion (BRI1-nYFP; BES1-nYFP) and pDEST-GW-VYCE (TdPP1-cYFP) for a C-terminal fusion with YFP [49]. *N. benthamiana* leaves were co-agroinfiltrated at an OD600 nm of 0.8 as previously described [47], with either TdPP1-cYFP and BRI1-nYFP, or TdPP1-cYFP and BES1-nYFP. Leaves were observed by confocal microscopy (Leica TCS SP5 II) two days after co-infiltration.

### 4.8. Real-Time Quantitative RT-PCR (qRT-PCR)

For real-time quantitative RT-PCR (qRT-PCR), seedlings were grown as described above and total RNA was extracted using the Plant RNA kit (OMEGA Bio-tek, Norcross, GA, USA). cDNA was synthesized using the iScriptTM gDNA clear cDNA Synthesis Kit (Bio-Rad) according to the manufacturer’s instructions. qPCR was performed in a BioRad CFX96 real-time system with HieffTM qPCR SYBR Green Master Mix (Yeason); 20 μL of the PCR reaction mixture contained 10 μL of SYBR Green mix (Bio-Rad, Hercules, CA, USA), 1 μL of primer mix (10 μM), 1 μL reverse-transcribed product and 8 μL of water. Details on gene-specific and normalizer (ACT2 and GAPC2, were used as normalizer) primers are indicated in Supplementary Table S1. Data were analyzed using the 2^−ΔΔCT^ method [50].

### 4.9. Confocal Imaging of Arabidopsis and N. Benthamiana

Confocal images were acquired using Leica TCS SP8 or Leica TCS SP5 II scanning confocal microscopes. To measure the meristem size, 5-day-old seedlings were fixed with 4% paraformaldehyde, then treated with ClearSee solution as previously described [51] and finally stained with calcofluor-white. The number of cortex cells in individual cell files was used to gauge the meristem size. The meristematic zone was defined as the region of isodiametric cells from the QC up to the cells that were twice longer than the immediately preceding cells. The settings used for the laser scanning are 405 nm excitation and detection at 425–475 nm for calcofluor-white and 514 nm excitation and detection at 525–570 nm for YFP. Images have been analyzed using Fiji [45].

### 4.10. Statistical Analyses

Statistical analyses were performed using GraphPad Prism6 software using *p*-values < 0.05 and Tukey’s multiple comparison test.

## Data Availability

No new data were created or analyzed in this study. Data sharing is not applicable to this article.

## Supplementary Materials

The following are available online at https://www.mdpi.com/article/10.3390/ijms221910424/s1, Figure S1: Elongation zones of *TdPP1* transgenics are hyposensitive to high concentration of exogenous eBL, Table S1: List of primers used in the present study.
