# Supplementary material for: Wheat Type One Protein Phosphatase Participates in the Brassinosteroid Control of Root Growth via Activation of BES1
Source: Int J Mol Sci. 2021 Sep 27;22(19):10424. doi: 10.3390/ijms221910424 (PMC8508605; doi:10.3390/ijms221910424)
Supplement: Supplementary file 1 [file ijms-22-10424-s001.zip › ijms-1337177-supplementary.pdf]

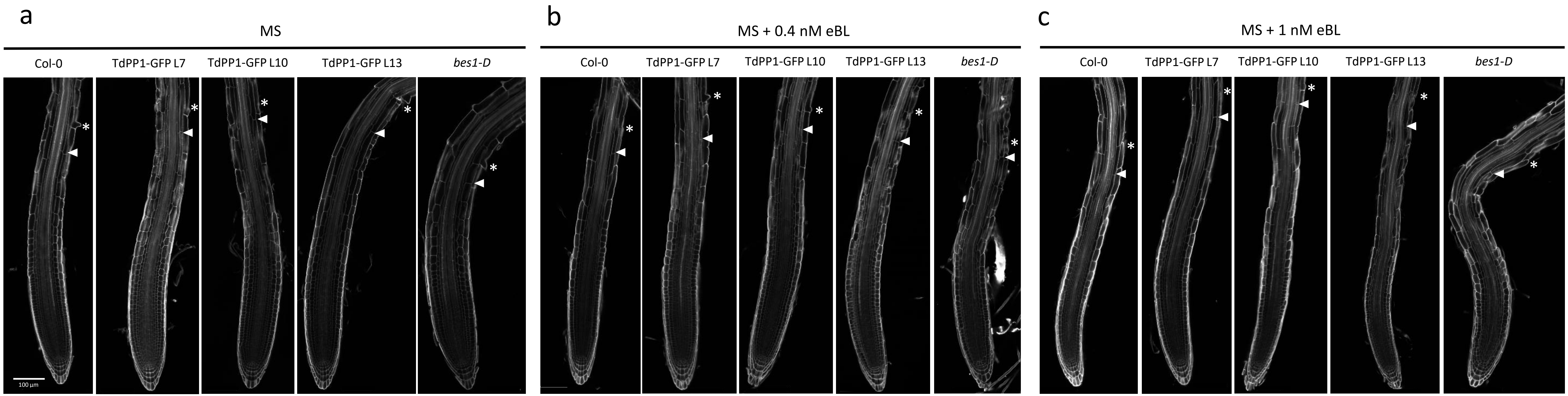

Supplementary Figure S1

Supplemental Table S1. List of primers used in the present study.

Description	Primer Name	Primer Sequence (5′→3′)
Real-time PCR primer for *TdPP1* ORF (KM203893)	qRT TdPP1-Fw	5′-ATGAATGCAAGCGTCGCTTC-3′
	qRT TdPP1-Rv	5′-ATCAATTAGAGCGGCCACAG-3′
Real-time PCR primer for *DWF4* (*At3g50660*)	qRT DWF4-Fw	5′-TGGCAACAGCAAAACAACGG-3′
	qRT DWF4-Rv	5′-ACGGCATGTAGTTGTTTCCC-3′
Real-time PCR primer for *CPD* (*At5g05690*)	qRT CPD-Fw	5′-TGAAACAACCTCCACGATCATGA-3′
	qRT CPD-Rv	5′- TGCCCTAATCTTTTCATGCTCTTC-3′
Real-time PCR primer for *Actin 2* (*At3g18780*)	qRT ACT2-Fw	5′-CAGTGTCTGGATCGGTGGTT-3′
	qRT ACT2-Rv	5′-TGAACGATTCCTGGACCTGC-3′
Real-time PCR primer for *GAPC2* (*At1g13440*)	qRT GAPC2-Fw	5′- TTTCCGTGTTCCAACCGTTG-3′
	qRT GAPC2-Rv	5′- AGATTCCTCCTTGATGGCCTTC-3′
